# PhenoImageShare: an image annotation and query infrastructure

**DOI:** 10.1186/s13326-016-0072-2

**Published:** 2016-06-07

**Authors:** Solomon Adebayo, Kenneth McLeod, Ilinca Tudose, David Osumi-Sutherland, Tony Burdett, Richard Baldock, Albert Burger, Helen Parkinson

**Affiliations:** MRC Human Genetics Unit, IGMM, University of Edinburgh, Crewe Road, Edinburgh, UK; Department of Computer Science, Heriot-Watt University, Edinburgh, UK; European Bioinformatics Institute (EMBL-EBI), European Molecular Biology Laboratory, Wellcome Trust Genome Campus, Hinxton, CB10 1SD UK

**Keywords:** Image annotation, Genotype-phenotype associations, Semantic annotation

## Abstract

**Background:**

High throughput imaging is now available to many groups and it is possible to generate a large quantity of high quality images quickly. Managing this data, consistently annotating it, or making it available to the community are all challenges that come with these methods.

**Results:**

PhenoImageShare provides an ontology-enabled lightweight image data query, annotation service and a single point of access backed by a Solr server for programmatic access to an integrated image collection enabling improved community access. PhenoImageShare also provides an easy to use online image annotation tool with functionality to draw regions of interest on images and to annotate them with terms from an autosuggest-enabled ontology-lookup widget. The provenance of each image, and annotation, is kept and links to original resources are provided. The semantic and intuitive search interface is species and imaging technology neutral. PhenoImageShare now provides access to annotation for over 100,000 images for 2 species.

**Conclusion:**

The PhenoImageShare platform provides underlying infrastructure for both programmatic access and user-facing tools for biologists enabling the query and annotation of federated images. PhenoImageShare is accessible online at http://www.phenoimageshare.org.

## Introduction

As reference genomes and large-scale programs to generate model organism mutants and knock-outs are completed, there have been parallel and complementary efforts from projects such as the International Mouse Phenotyping Consortium (IMPC) and the Asian Mouse Phenotyping Consortium (AMPC) to establish and codify phenotype with genomic coverage [1]. Current phenotyping efforts typically deliver a variety of images with annotations describing the phenotype on display. These are then stored in independent databases associated with the primary data. These databases may be searched individually, albeit there is no mechanism for integration, cross-query or analysis, especially with respect to human abnormality and disease phenotypes. We have developed PhenoImageShare (PhIS) to address this problem. PhIS is a cross-browser, cross-repository platform enabling semantic discovery, phenotypic browsing and annotation of federated phenotypic images. PhIS provides a centralised repository that stores a limited set of meta-data including links to the originating resource, and the annotations generated through the use of PhIS. As such, the “complete” PhIS system is a federated resource that includes the central PhIS database and the repositories of the underlying image sources.

Resources such as OMERO [2] allow users to store their images, but do not provide ontology-enabled tools. Further, images are often “siloed” by imaging methodology or domain [3] and access to multiple different image repositories may require bespoke development against multiple modes of programmatic access which may evolve as resources change. PhIS achieves this level of integration by using a lightweight annotation document structure, which can then be exposed through standard query engines such as Solr [4]. PhIS is species and imaging technology neutral and currently provides access to 117,982 images federated from four different data resources with 53,000 regions of interest (ROI) associated to anatomy or phenotype ontology term annotations. These can be accessed via the web GUI or programmatically via web services. To date PhIS is populated with images from Drosophila and three different mouse projects.

### Related work

Whilst no existing service or tool is directly comparable to PhIS a number of products may be considered similar. The Yale Image Finder (YIF) [5] offers an interface to query over 1.5 million images from open access journals. A user has a few options to restrict or loosen the search to image description, whole article or abstract. NCI Visuals Online [6] provides access to about 3000 images with the possibility to search the image descriptions making use of common text-search functionality such as quotations for exact matches, term exclusion, multiple keywords, defaults to case insensitive search, stemming, et cetera. These are useful resources that address a different set of needs from PhIS. Ontology-backed search (i.e. semantic search) is not supported, although an advanced text search exists. YIF does not support the display of Regions Of Interest (ROI), online annotation, image submission or resource integration via an API.

The Semantic Enrichment of Biomedical Images (SEBI) [7] semantically enriches image meta-data from YIF and enables search over that meta-data. The SEBI platform is based upon a number of different modules. Collectively these modules facilitate automatic annotation (i.e., semantic enrichment) by using Semantic Automated Discovery and Integration (SADI) [8] enabled services to pull related information from other resources. When automatic annotation fails, there is provision for crowd-based annotation. The generated data is stored within a triplestore called iCryus [9] that can be queried through a SPARQL endpoint or navigated via a RDF browser. Both SEBI and iCyrus focus on DNA/protein sequence images rather than the phenotypic images found within PhIS. Another difference is the approach to annotation creation. SEBI/iCyrus take the meta-data associated with an image and extend it by using other services available on the Semantic Web. PhIS operates at a different level, helping to create and publish human-expert-generated annotations. A SEBI/iCyrus-like platform for phenotype images would be complementary to PhIS. An iCyrus-like phenotoype tool would pull image data from PhIS in same way that iCyrus pulls data from YIF.

Another framework is proposed by Kurtz et al. [10], with support for annotation suggestion and semantic annotations, with focus on similarity metrics but with no link to an application.

Wang et al. [11] have addressed the need for an ontology-assisted image-annotation tool and have produced software that supports RadLex [12] backed annotation of radiology images. While the project was successful, its scope is restricted to radiology annotations and is not accessible online to external users. It does not aim to offer further integration with other resources through any API.

A set of commercial desktop applications such as Osirix [13] and Amira [14] offer segmentation assistance and plain-text annotations, either integrated or through plugins. AISO [15] is a desktop tool that was developed for plant image annotation and supports ontology use. AISO is restricted to the plant ontology, which it accesses through its own web service. OMERO offers annotation support both on the web client and the desktop application but no ontologies are integrated. The OMERO server is not an online service but a server distribution so every group or institution needs to host its own server. PhenoImageShare is a complementary resource that can be used in association with OMERO hosted images.

There is a wide variety of public image portals [3] with some focusing on phenotype images: IMPC portal, EMAGE [16, 17], The Cancer Genome Atlas [18] to mention a few. All of these focus on species- or project-specific images and image submission is not open to the outside user. IMPC and EMAGE support ontology annotation but have limited or no support for region of interest (ROI) display on the original image, however EMAGE does support spatial mapping onto a standard atlas for the purposes of spatial comparison and query.

We have identified the need for a public and easy to use web service that can federate cross-species, cross-project images, with open image submission and powerful semantic search. There is also a clear need for an online image annotation tool with support of ontology terms and different ontologies. To the best of our knowledge such a tool does not exist.

## Methods

**Fig. 1:**
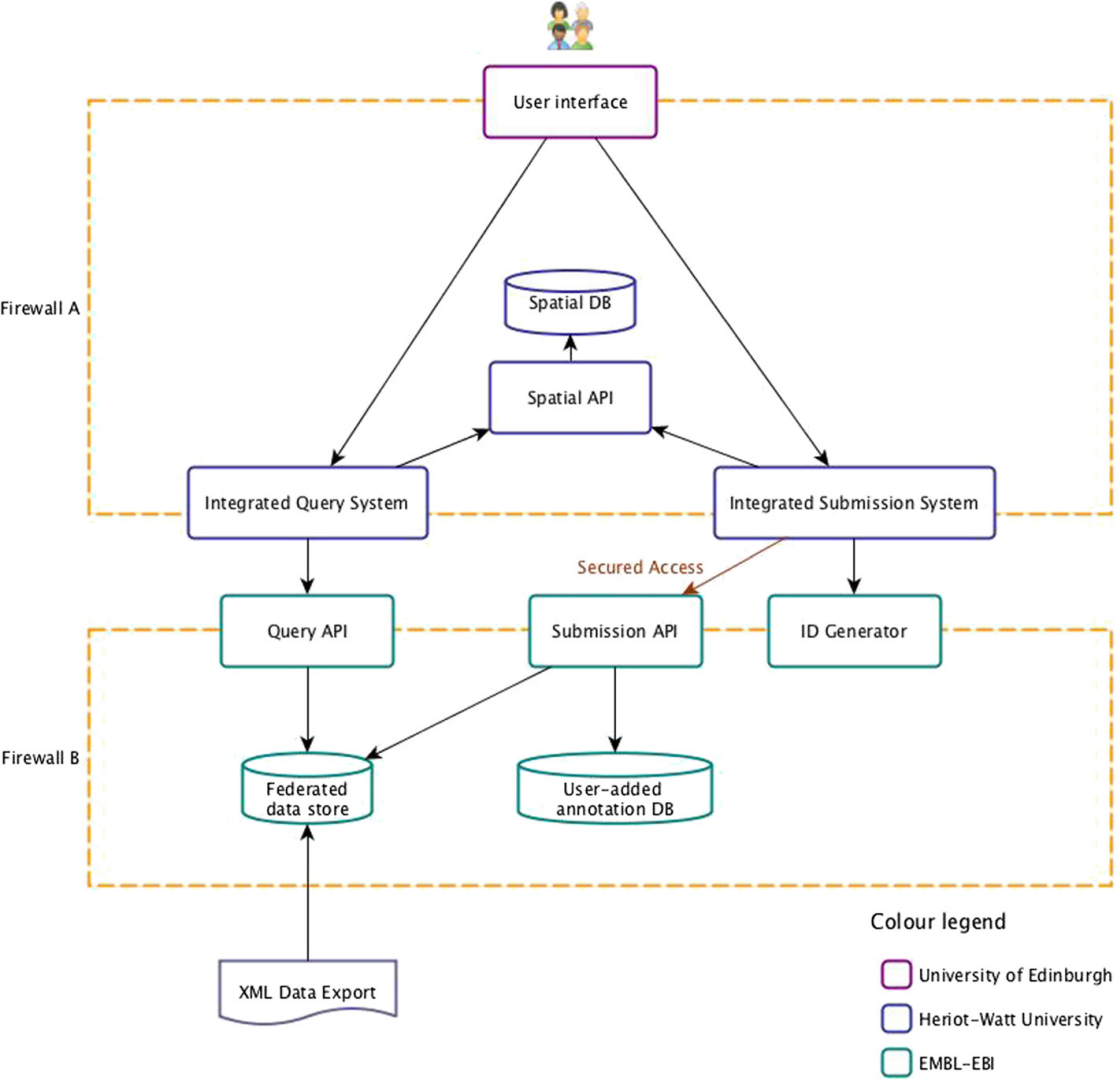
PhIS architecture. The different software components part of the PhIS platform. The *arrows* describe the collaboration of the components and the direction of the function calls. For example the UI uses the methods provided by the IQS but the IQS alone cannot trigger any actions in the UI. The annotation submission API needs to be secured in order to keep the integrity of the database but the query API is not restricted

The PhIS platform consists of three main software layers: the User Interface (UI), the integration layer and the backend services. The UI components provide an intuitive Graphical User Interface (GUI) to query cross-platform image meta-data. The GUI also provides a basic ontology-enabled annotation service to allow image owners, and third parties to annotate images using ontologies for their own use and also for sharing images and annotations between consortia and collaborators. The integration layer is the one which consolidates access to the different backend services into one access point, used by the GUI. The backend services provide API methods to query and annotate the data as well as a data import mechanism. The architecture described is also represented in Fig. 1.

### Image discovery infrastructure

The query API offers a simple REST-like interface to access the data in multiple ways. Approximate search methods are provided with the purpose of image discovery but also specific interfaces for example “search by id” are available. The API provides an endpoint for query-suggestion options on an input string. This was designed to follow the following order: 1) exact matches, 2) phrases that start with the given string as a word, 3) phrases that start with the given string as part of the first word, 4) exact match in a word in the phrase, other than at the start of it, 5) matches where another word in the phrase starts with the given string.To illustrate this through an example, for the string *“eye”*, the suggestions will come in the following order: *“eye”* (Case 1), *“eye hemorrhage”* (Case 2), *“eyelids fail to open”* (Case 3), *“TS20 eye”, “TS21 eye”, “left eye”, “right eye”, “narrow eye opening”, “abnormal eye development”* (Case 4), *“abnormal eyelid aperture”* (Case 5). We have implemented this after a series of formative sessions with users. We achieve this sorting by applying different boost factors to Solr text fields tokenized in different ways. Text matching is case insensitive in all cases.

**Fig. 2:**
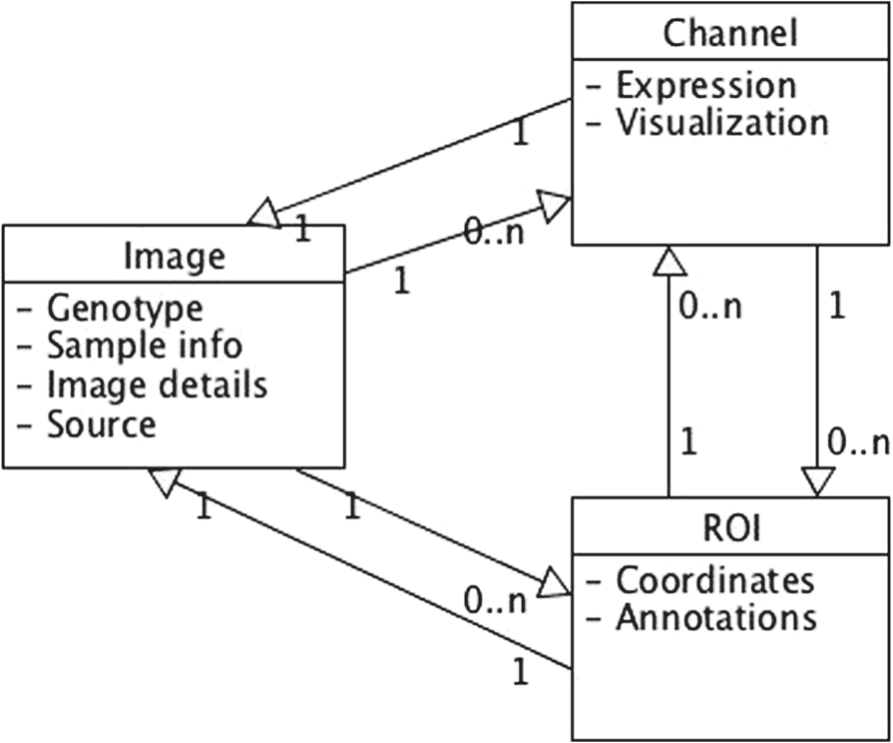
PhIS schema. The image entity is the core meta-data object and stores generic, immutable meta-data such as imaging procedure and details, data source, credits and sample information, genotype or sample preparation techniques. The channel entity stores visualization information, such as tags or labels and genomic information when needed, such as for expression images. The ROI entity holds both the coordinates, within the given image, and the annotation values

The database, XML schema (XSD) and Solr representations share a common high-level schema such that the information storage is split three ways: image information, channel information and ROI/annotation information. This is represented in a simplified way in Fig. 2. Provenance information is an important part of the schema. PhIS currently implements a pragmatic simple model, covering image/annotation creator, annotation editor, image and annotation creation/last edit date, repository or resource of origin or publication if one is provided for the image.

The API offers JSON responses, which is the standard for modern APIs, is straight forward to consume by the UI, has the advantage of human readability and is also the best fit for our Solr-backed infrastructure. As the API gains traction and users require other formats, new response types can be evaluated and provided. The approach used to create the data model for the Annotation and Image Markup project [20] has been applied to PhIS. The implementation is in Java and the query functionality is primarily via Solr. Solr is built upon Lucene [19], which is an open source indexing and search engine. Lucene-based technology powers a number of existing image repositories, e.g., OMERO and Yale Image Finder both use Lucene to search for images. The authors have experience of using Solr to build image repositories from their work on the IMPC portal. Solr offers fast, flexible, robust, open source search solutions with scaling options such as Solr cloud. Through a varied array of features Solr enabled us to build a rich search and navigation experience with little resources.

### Spatial annotations

Many phenotypes occur in a particular spatial location, which can be described using either an anatomy (ontology) term or by being mapped onto a biomedical atlas [20]. Currently, PhIS only supports anatomy-based descriptions, with atlas-based descriptions targeted for a future release. Anatomy-based descriptions are simpler to use, but less precise.

Should a PhIS submission be missing a spatial description, it may be possible to infer an appropriate anatomy term from a supplied phenotype term. For example, given the phenotype term “cataract” the anatomy term “eye” can be inferred. This inference relies upon the bridge ontologies provided by the Monarch initiative [21]. Inferred spatial annotations are pre-computed and stored within Solr allowing them to be queried by a user.

### User interface

PhIS delivers its functionality through a highly responsive and easy-to-use web interface built upon standard technologies such as Python, Django, JavaScript, Asynchronous JavaScript (AJAX), Bootstrap [22] and Flat-UI [23]. Portability, reusability and responsiveness are at the core of GUI design considerations.

Throughout the development of PhIS a series of *formative* evaluation sessions have been used to determine and then prioritise requirements. This included the creation of a series of tasks against which the functionality of the search capability can be tested. Additionally the search functionality has undergone a small scale *summative* evaluation. Feedback from this has been integrated within the current beta release. Development of the annotation tool started after the search functionality, and so the annotation tool is still undergoing an iterative process of formative evaluation and development. A comprehensive summative evaluation that tests the complete workflow (search and annotation) will be undertaken when the annotation tool reaches maturity.

There are four distinct elements to the PhIS GUI, and this section discusses each one in the order a user encounters them in a typical workflow.

#### Landing page

When arriving at www.phenoimageshare.org a user is greeted by a visual summary of the data held within the PhIS repository that sits below a search box. The visual summary consists of three pie charts that collectively quantify and classify the types of images stored. The three dimensions visualised are imaging method, sample type (mutant vs. wild type) and image type (expression vs. phenotype). Clicking on a block within one of the charts performs a query. For example, clicking on the *macroscopy* block will display all the images that have meta-data indicating they are macroscopy images.

Alternatively, the user can use the search box to query PhIS. A query may consist of free-text, gene or allele symbols, anatomical or phenotypic terms from standard ontologies. An query-suggestion facility integrated within the search box provides the user with a drop-down list of terms predicted from the set of existing annotations. Search results are displayed in real-time on a dedicated page.

#### Search interface

**Fig. 3:**
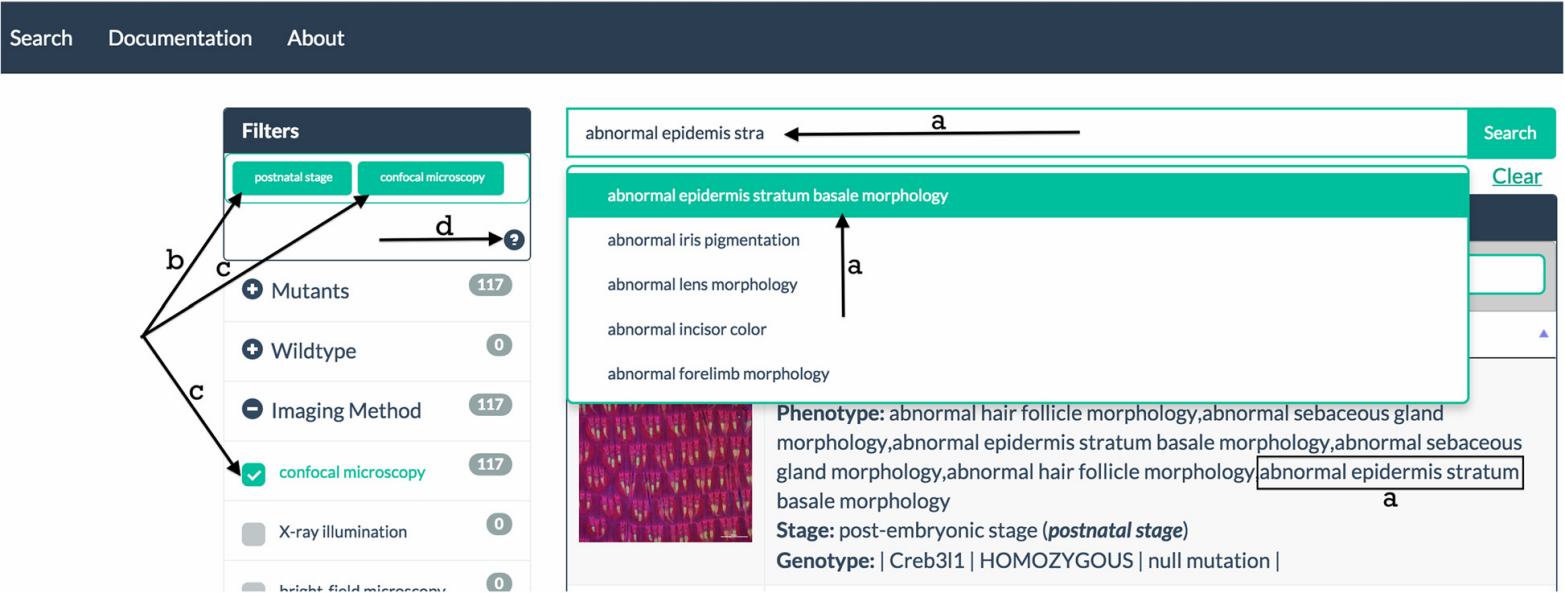
Query interface with ontology-based search and faceting functionality. Search results for a phenotype (abnormal epidermis stratum basale morphology) (**a**) is filtered by development stage (postnatal) (**b**) and confocal microscopy imaging method (**c**). Example query and help on using facets (**d**) are also provided

This page (see Fig. 3) displays a list of images that meet the user’s query criteria, and provides the ability to filter that list or navigate through it. For each image in the list a brief summary of the image’s meta-data is displayed alongside a thumbnail.

To reduce the images returned by a query the user can apply a range of predefined filters or a faceted search. Both the filters and the search are facet-based and meta-data driven. Nested checkboxes allow the user to reduce the images displayed using filters including imaging method, stage, species and resource (i.e., the online resource from which PhIS obtained the image). Faceted search is available for anatomy, gene/allele and phenotype. If the user searches for ’heart’ in the anatomy facet search box, only those images from the original query with an anatomy annotation featuring the term ’heart’ will be displayed. The filters and faceted search can be combined to deliver powerful customised queries.

To find an image of interest the user can either adjust the filters/search or (s)he can undertake a completely new query using the main search box (now in the menu bar). When the user identifies an image of interest in the search results, clicking on that image reveals more information in the Image Interface page.

#### Image interface

**Fig. 4:**
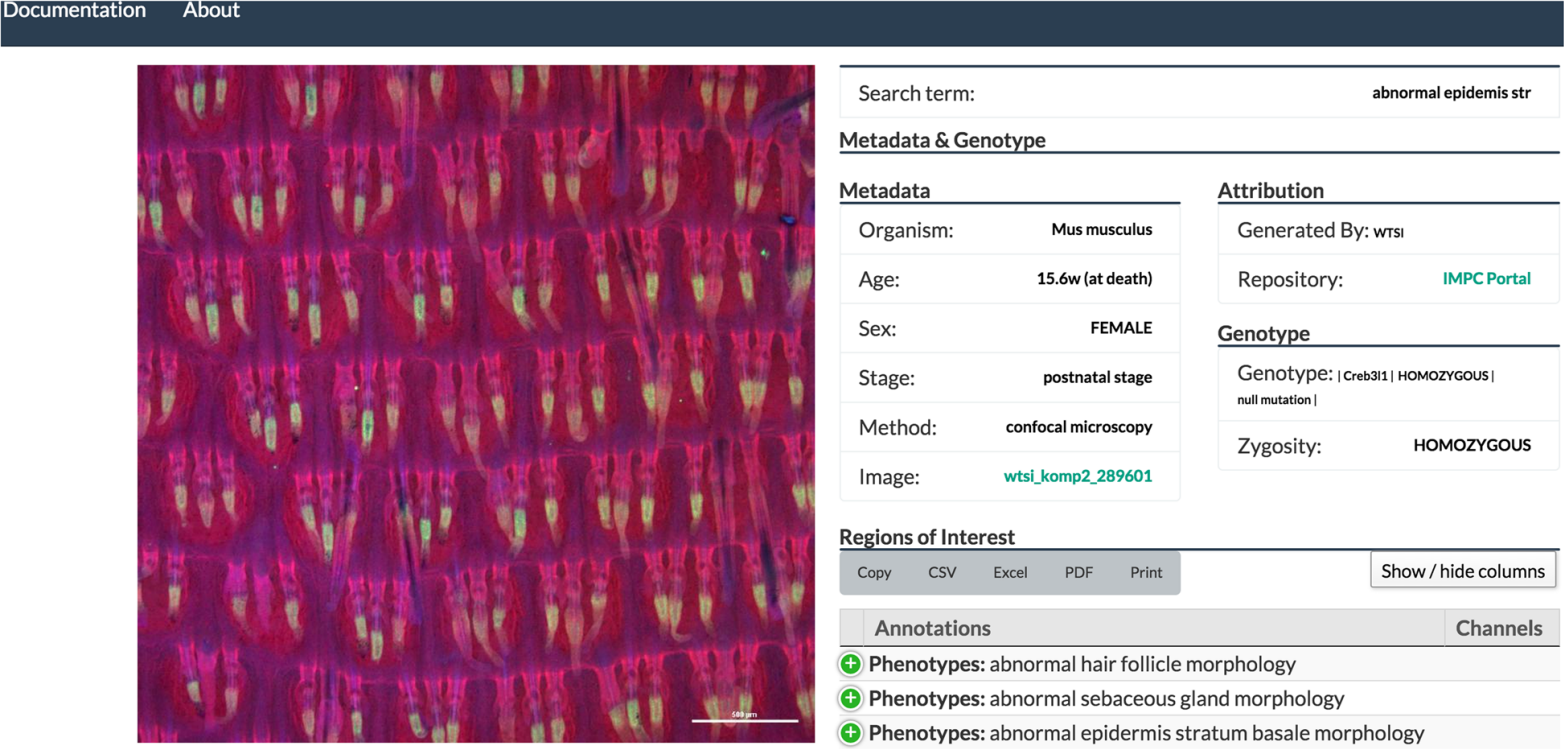
Detail view, showing meta-data, provenance and annotations associated with one of the **images** returned in the search shown in Fig. 2

In the Image Interface (see Fig. 4) a single image is displayed alongside a more comprehensive set of meta-data for the image. Meta-data shown includes data captured from the source (e.g., genotype, phenotype and provenance) and annotations generated by users of PhIS. The meta-data contained within PhIS is a subset of the data available at the originating resource, thus PhIS provides a link back to the originating resource enabling the user to investigate further.

Image annotations can be exported in a variety of formats, with new formats to be added. The user can add their own annotation to this image using the PhIS annotation tool.

#### Annotation interface

The annotation interface allows the users to semantically annotate an image. Annotations can apply to the whole image or to a region of interest within the image. ROIs are indicated by the user drawing a rectangle upon the image. This rectangle is treated as part of the annotation and is stored within PhIS for later reproduction. When creating an annotation, the user has the ability to select a series of ontology terms obtained through the BioPortal widget [24]. All annotations submitted through the PhIS interface will appear on the website and become searchable instantly.

Currently the annotation tool only supports low resolution images; however, it has been implemented with the goal of displaying high resolution images via OpenSeadragon [25]. OpenSeadragon is an extensible JavaScript library providing high quality zooming functionality that will enable PhIS to support high resolution images. Because it works well with OpenSeadragon, and required minimal extension, FabricJS [26] provides the functionality for drawing ROIs on the images. Other possible options (e.g., Annotorious [27] or AnnotatorJS [28]) were rejected because their support for OpenSeadragon was too immature at the time of testing.

**Fig. 5:**
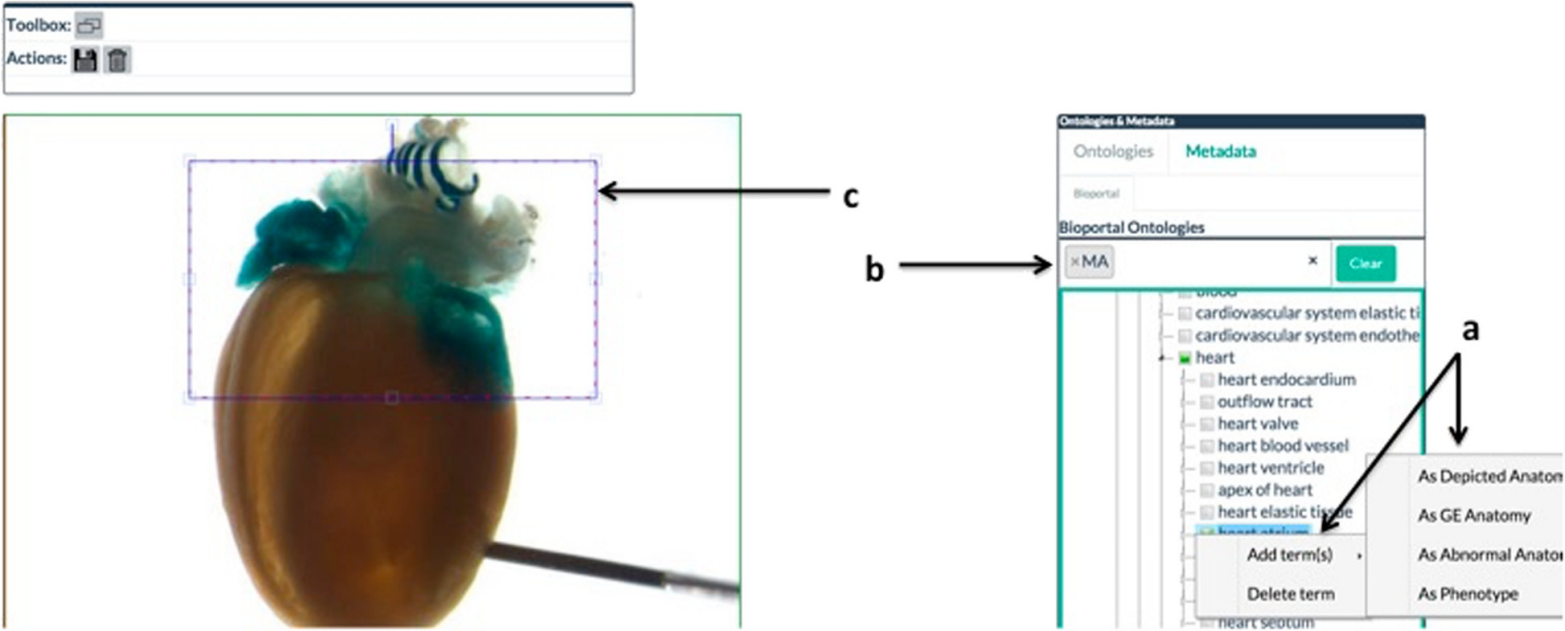
Annotation interface in edit mode. Context menu (**a**) is available to user via right-click on selected terms from an ontology (**b**). User appends selected ontology terms to the drawn region of interest (**c**)

Figure 5 presents a screenshot of the Annotation Interface in use.

## Data and ontologies

To make data available through PhenoImageShare batch submissions are generated in XML format by the resources wishing to submit data to PhIS. Our data releases are versioned and the current one is listed in the lower right corner of the page, which links to more details about each release. Old releases can be accessed programmatically, through the API. Data sources provide PhenoImageShare with up-to-date XML exports of their data, following the PhIS schema (XSD). Producing the XML will vary based on the amount of data exported while the processing on the PhIS server only takes a few minutes. However, we do data releases so there might be a few weeks lag between the moment the export is ready and the moment the data is published live. It is up to the data submitter how often updated XML exports are submitted. We then perform XML and semantic validation, followed by data enrichment before indexing. This includes addition of synonyms and ancestor relationships to the Solr index allowing an improved semantic search. Some examples of semantic validation include checking if the term provided as an anatomy annotation comes from an anatomy ontology or checking if the label provided with the ontology id matches. Ontology curators sometimes update the preferred labels and databases have trouble keeping up with that. As part of our import process we select the most up to date label and index the old one as a synonym. If the ontology term was deprecated and a replacement one is suggested we use the replacement term. In order to achieve this semantic manipulation of the data we make extensive use of the OWL API [29] and the Elk reasoner [30] to precompute relations that we then index in Solr. This approach offers more restricted semantic capabilities than RDF/SPARQL, but it is faster to set up and covers our use-case needs, both in terms of semantic and text-based search capabilities.

### Data

The current PhIS release contains data from four sources, presented with more details in Table 1.

- The TRACER database [31] contains imaging for embryos carrying a transgenic insertion generated by the Sleeping Beauty transposon-based system.

**Table 1:** PhenoImageShare data

Resource	Imported images	Life stages	Image types	Specie	Main annotation type
WTSI KOMP2	93861	Embryo, adult	X-ray, macro photographs,	Mus musculus	Ontological
			histopathology, lacZ expression		
TRACER	702	Embryo	Expression	Mus musculus	Controlled
					vocabulary
EMAGE	3566	Embryo	Expression	Mus musculus	Ontological
VFB	19853	Adult	Expression	Drosophila	Ontological
				melanogaster
- Images from the Wellcome Trust Sanger Institute, generated as part of KOMP2 [32] focus on single gene knock-outs. Genotype, anatomy and phenotype annotations are provided for these data.
- The EMAGE database provides embryo gene expression images.
- Virtual Fly Brain (VFB) [33]. The VFB dataset offers an interesting use-case for cross species integration, but also adds value to our anatomy coverage with its focus on neuroanatomy and gene expression in the adult Drosophila melanogaster brain.

### Ontologies

To add semantic value (i.e. synonyms, hierarchical relations, ontology cross references) and make the integration possible we support the use of different ontologies. For anatomy annotations we use MA, EMAP, EMAPA [34, 35] and FBbt. For cross species integration we make use of UBERON [36] and the Monarch Initiative’s bridge files. We also have phenotype terms currently from MP [37] and we expect to add further ontologies for pathology, and other species in future releases. Other ontologies critical for the development of PhIS are the species specific developmental stage ontologies (Mmusdv, FBdv) and the Fbbi ontology for biological imaging methods, originally developed for the Cell Image Library [38]. There has been an increase in the effort of building and using biomedical imaging ontologies in the recent years. Some of these ontologies and terminologies are reviewed in a recent survey [39] and we have evaluated them with respect to our needs as well. Fbbi had the best term coverage for our initial use-cases. BIM [40] for example has about 100 terms (classes) whereas Fbbi covers about 600. However, PhenoImageShare is not tied to any set of ontologies and the ones mentioned above are merely the ones used in the existing dataset. Any ontology distributed in an OWL/OBO format can be included.

Because we integrate data from different species and annotated with different ontologies, common higher level terms to display as facets are unavailable in the species specific ontologies used. To overcome this we use generic ontologies such as UBERON. For example, the developmental stage terms we have at the moment are quite varied and come from two different ontologies, which are not directly compatible. We make use of the cross references and thus are able to map the species specific term to the generic UBERON term, which we then use to filter the data. Filtering for *“embryo stage”* (UBERON) will display images for mouse embryonic stages, e.g. *“Theiler stage 18”* but also images for prenatal stages in other species, e.g. *“gastrula stage”* in fly, if available. The section on spatial reasoning describes an example of how we infer anatomy terms from phenotype terms.

## Discussion

Leveraging the semantic options provided by the use of ontologies, PhIS is able to offer a better search experience as well as a simplified display, for example by grouping filter options by top-level terms or some higher level terms. The same is available in a behind the scenes way through the API. When searching for “cardiovascular”, the first images coming up are the ones directly annotated with terms containing “cardiovascular”. These are followed by images annotated with parts of the cardiovascular system, i.e. “blood vessel”, “heart”.

Currently, PhIS facilitates a basic spatial search by using the part-of relationship. The components of a particular structure are returned when the structure is queried, e.g., if “brain” is the query then images annotated with the term “diencephalon” will also be returned. As outlined in [41] efforts are ongoing to include further spatial relationships within PhIS’s search capabilities. Relationships are sourced from the Biological Spatial Ontology [42] and include left-of and right-of. These relationships will enable PhIS to return images that have annotations featuring anatomical structures that are adjacent to (immediately right-/left-of) the query term. For example searching on “spleen” may return images annotated with “stomach” because the stomach is immediately right of the spleen. Adding relationships for dorsal/ventral to and cranial/caudal to will provide a more complete notion of adjacent. As with part-of, the ordering of the results will be important and this needs further exploration. The power of using spatial relationships extends to the case of tissue or structural classes and enables query in terms of similar tissue relationships for example the search “next-to joint” queries for a tissue adjacent to a joint without needing to specify which joint. There are of course limitations so a search on “adjacent-to muscle” is unlikely to be sufficiently specific to be useful but the extension to spatial search is powerful and complementary to the standard search capabilities of PhIS.

### Use-cases

Images are critically important to biologists trying to build a clear picture of the nature of phenotypes and expression patterns. This clear picture can be essential to researchers aiming to find an experimental marker of some specific tissue type or generating hypotheses about the function of a gene, structure or functioning of some specific anatomical entity.

Increasingly, such image data is contained in bulk image data-sets, where it is hard to search. PhenoImageShare provides biologists with simple, intuitive ways to find images of phenotypes and expression patterns from bulk image datasets that would otherwise be very difficult to search. A single interface allows semantically-enhanced searches across multiple datasets from a range of biologically-relevant starting points. For example, a researcher can search with a gene name to find images of mutant phenotypes and expression patterns for that gene and then narrow the set of images displayed to those in some relevant class of anatomical structure, or to those with some specified imaging modality. Or they can search with an anatomical term, then narrow their search with additional anatomical terms or imaging modalities. Semantic enrichment via ontologies means that users do not need to know the precise terms used in annotation. Searching for bone, for example, pulls back images annotated with the term rib.

Our current image use-cases have been developed from the data immediately available and are examples of 2D image data, from a variety of sources specified for the first phase of the project and selected to illustrate the capabilities of the system.

In terms of annotations, the first use-case is drawing basic ROIs shapes with the ability to annotate them with ontology terms.

From the point of view of an image provider, PhIS offers several benefits. One of them is a simple mechanism to upload image annotations and have them online, easily accessible and discoverable. The goal of PhIS is to create a central source from which users can search for, or navigate to, the phenotype image they need. If a critical mass is reached, such a central source would become the go-to venue for phenotypic images. Because PhIS links to the underlying resources that contribute images, it can greatly increase the discoverability of many image resources and thus increase the number of end-users for those resources. Additionally, PhIS provides image resources with the ability to annotate their images without developing or installing additional tools. The query API could greatly simplify the development of a project-specific website.

### Future work

Features to be supported in future releases include query sharing and bookmarking, image discovery by similarities, complex query builder, links to reference atlas frameworks such as EMAP [17], data export in RDF format and analytics. A provenance ontology such as PROV-O [43] should be used to map our provenance annotations to ontology-defined data properties for an RDF export. The annotation tool will support hierarchical annotations, support for 3D image annotation and more shapes denoting ROIs and annotations. Other future infrastructural plans include offering the PhIS services as portable widgets such as those offered by BioJS [44] for inclusion in third party tools.

We also aim to extended the supported image types in the near term to large-scale histo-pathology images (2D) with complex cellular phenotypes and to 3D data captured as part of the Wellcome-funded Deciphering Models of Developmental Disorder [45] project. The extension to large-scale data will be coupled with the extension of the annotation model to allow mapping or registration of the image to a standard spatial framework, for example the models provided by the Edinburgh Mouse Atlas Project (EMAP) for the developing mouse embryo [16].

The mouse embryo use-case taps into a rich atlas-based spatio-temporal framework, which will allow testing of different types of spatial annotation. These range from the currently implemented within-image association of location (e.g. a ROI) with anatomical terms as described above through to full image mapping onto a standard 2D/3D atlas. Mapped data can then be used to infer co-location (co-registration) of image data and more interestingly other spatial relationships such as proximity, biological direction, connectivity and pattern similarity.

### Availability of data and materials

The data available in PhIS can be accessed via the web GUI or programmatically via web services. All code is open source and available from https://github.com/PhenoImageShare (back-end infrastructure code) and https://github.com/ma-tech/PhenoImageShare (user interface code). The code is released under the Apache 2.0 license. The query API is available and documented at www.phenoimageshare.org/data/rest/. The PhenoImageShare website is available at www.phenoimageshare.org.

## Conclusion

The PhenoImageShare platform provides underlying infrastructure for both programmatic access and user-facing tools for biologists enabling the query and annotation of federated images. It supports the use of ontologies and builds on these to deliver the best search experience and spatial reasoning. The release of an ontology-enabled annotation tool for image generators and third parties will allow projects such as IMPC to federate image annotation tasks and will provide a collaboration platform for annotators. The query API is exposed for programmatic access such that development against the PhIS API could be easily imagined, either for advanced query options or for integration purposes in other resources. We encourage owners and generators of image datasets to expose their data via PhIS and view it as sustainable platform for the sharing of image data, which requires minimal investment in disk, and supports a federated model of image data sharing.
